# Floating clavicle after a high velocity biking accident: A case report of an acromioclavicular dislocation with simultaneous proximal clavicle fracture managed surgically

**DOI:** 10.1016/j.ijscr.2021.106115

**Published:** 2021-06-16

**Authors:** Irene Blanca Moreno-Fenoll, Homero Valencia, Homid Fahandezh-Saddi, Elsa Arruti

**Affiliations:** Department of Orthopedic Surgery, Hospital Universitario Fundación Alcorcón, Budapest 1, E-28922 Alcorcón, Spain

**Keywords:** ER, Emergency Room, AP view, anteroposterior view, CT scan, Computed Tomography scan, ACJ, acromiocavicular joint, Floating clavicle, Bipolar clavicle dislocation, Panclavicular dislocation, Acromioclavicular joint, Sternoclavicular joint

## Abstract

**Introduction:**

Clavicle fractures and acromioclavicular joint dislocations are very common injuries. However, the combination of both, known as “floating clavicle” is extremely rare, with approximately 40 cases reported.

**Presentation of case:**

We report a case of a healthy 51-year-old male who suffered a high-velocity biking accident, with a bipolar clavicle injury (type IV acromioclavicular joint dislocation and proximal clavicle fracture), with concomitant rib fractures and pulmonary contusion. He received early surgical treatment by open reduction and osteosynthesis of the proximal clavicle (distal ulna plate, *Protean*®) and open reduction and stabilization with a *MINAR*® implant for the acromioclavicular joint. After an initial one-month immobilization, he started physical therapy. In the 10-month follow-up he presented with a pain-free full range of motion, a good cosmetic result, and radiological consolidation.

**Discussion:**

Bipolar clavicle injury is a rare clinical entity that encompasses a spectrum of combined clavicle fractures, acromioclavicular or sternoclavicular joint dislocations. They are sustained in a high-energy context, and accompanying injuries must be sought. Diagnosis is made through X-Ray and CT. Despite the lack of clinical guidelines, most authors agree on surgical management of at least one of the injuries, with multiple surgical techniques available. There is an emphasis in surgical treatment of the young and active patient. Conservative treatment is associated with poorer results.

**Conclusion:**

It is advisable to have a high index of suspicion for floating clavicle in a high-energy trauma patient, given possible life-threatening injuries, and long-term shoulder sequelae. Surgery should be considered in a young and active patient.

## Highlights

• “Floating clavicle” is a rare injury that should be suspected in a high energy accident.
• Other life-threatening injuries must be ruled out.
• There are no guidelines regarding best treatment.
• Surgical treatment is usually advocated for young and active patients.
• ORIF of sternoclavicular fracture and acromioclavicular joint dislocation yields good results.

## 1 Introduction

Clavicle fractures are common (2,5–4% of total body fractures in adults) [1], and so are acromioclavicular joint dislocations (9% of shoulder girdle injuries) [2]. However, the combination of both is extremely rare, with approximately 40 cases reported in the literature [3]. This clinical entity has been termed “floating clavicle”.

## 2 Case presentation

This case follows 2020 SCARE guidelines for reporting of cases in surgery [4]. A 51-year-old male, with no relevant medical-surgical history, right-handed, was referred to our hospital following clinical stabilization in the main Trauma Center of our area, where he had received initial treatment the previous day, after he had suffered a high-velocity biking accident. He was diagnosed with a right clavicle fracture, with sternoclavicular joint involvement. He had simultaneously sustained multiple rib fractures (2nd and 3rd right ribs), as well as a right upper lobe pulmonary contusion, with concomitant pleural effusion. The patient was clinically stable. Upon physical examination, he presented with pain and deformity at the proximal and distal end of his right clavicle, with a clear piano key sign at the acromioclavicular joint. Shoulder range of movement was limited by pain. Neurovascular examination of the extremity showed no deficits.

**Fig. 1:**
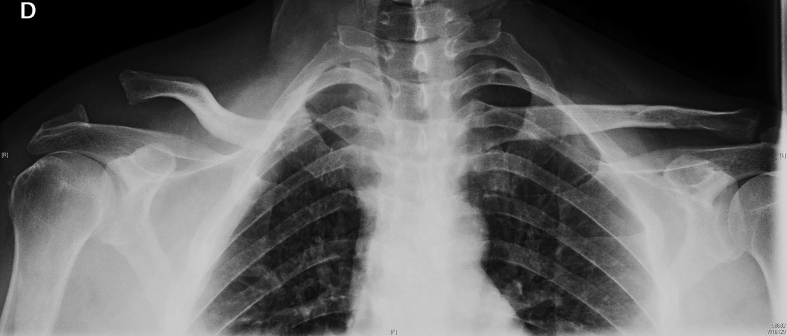
Initial AP X-ray that shows a Rockwood type IV acromioclavicular joint dislocation, and possible implication of sternoclavicular joint.

**Fig. 2:**
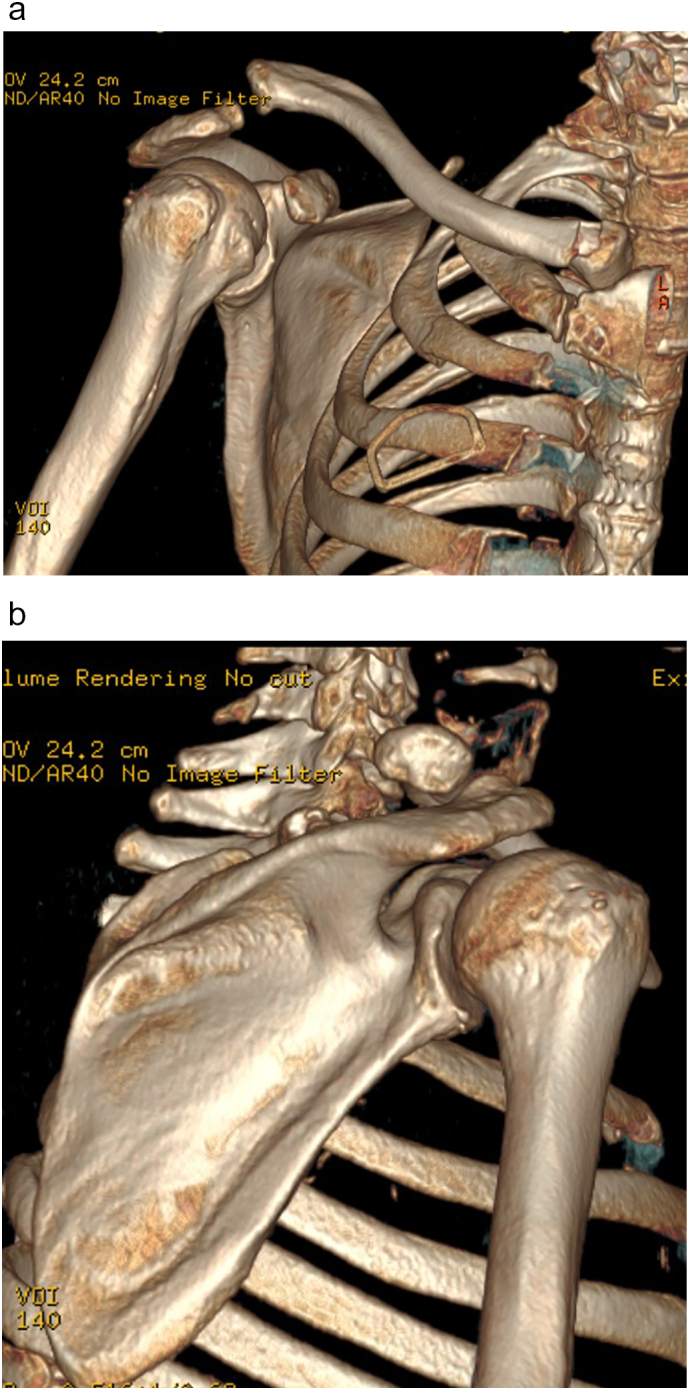
Computed tomography scan 3D reconstruction confirming a proximal clavicle conminuted fracture and ipsilateral 2nd and 3rd rib fractures (A) and posterosuperior displacement of clavicle at the acromioclavicular joint (B).

On the simple X-ray a Rockwood type IV acromioclavicular joint dislocation was detected. There was a suspected Allman type III fracture of the proximal clavicle, with an approximate shortening of 1.8 cm, shown on a full-chest X-ray, allowing comparison with contralateral extremity (Fig. 1). The study was completed by a computed tomography scan and 3D reconstruction of the clavicle, which confirmed an upward and backward displacement of the lateral end of the clavicle (11 and 6 mm, respectively), and a medial end comminute and extraarticular fracture (Fig. 2). This confirmed clinical suspicion of floating clavicle.

Surgery was performed by the authors (*H.V.G.*, *H.F.D*, *I.B.M.F.*, *E.A.P*) five days after the accident, with a stepwise approach of both injuries. First, through a medial horizontal approach, proximal clavicle was reduced and fixed with a distal ulna plate (*Protean*®, Skeletal Dynamics). Second, through a lateral saber incision, the acromioclavicular joint was reduced and stabilized by means of a *MINAR*® implant, a double supraclavicular and subcoracoid button system.

**Fig. 3:**
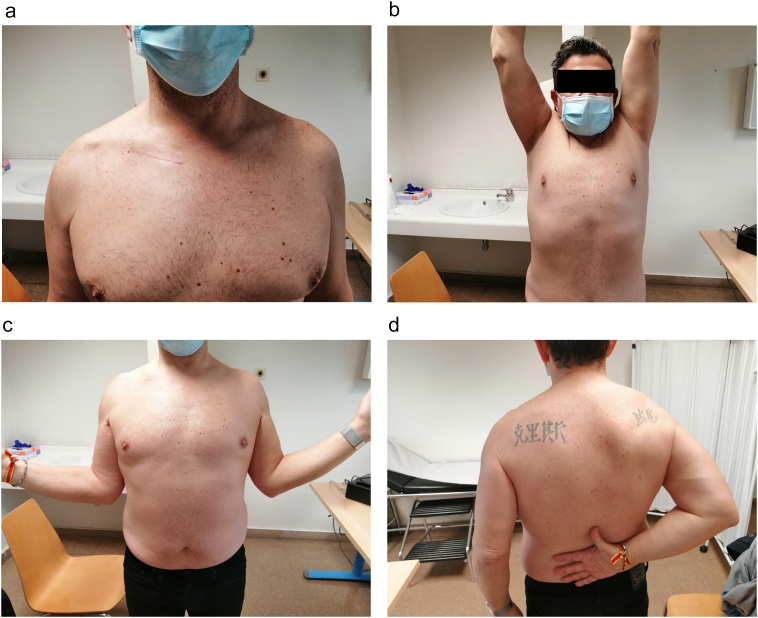
Clinical outcomes at 4-month follow-up, showing a good cosmetic result (A) and complete range of movement (B, C, D).

**Fig. 4:**
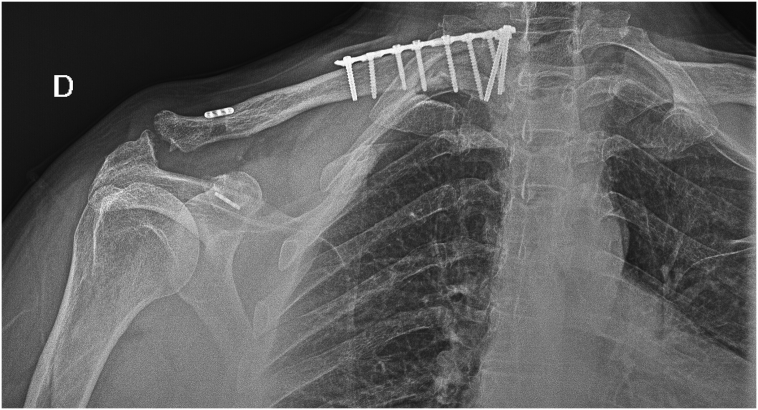
Radiological control at 4-month follow-up, showing proximal clavicle consolidation and good reduction of acromioclavicular joint.

An initial 4-week sling immobilization regime was started, allowing only elbow flexion extension and shoulder short-range pendulum movements. Within the first month, free shoulder motion was allowed, and the patient started physical therapy. In the second postoperative month, he returned to progressive sports practice, with elastic-band exercises and running. In the 4-month follow-up he presented with a pain-free full range of motion, and a good cosmetic result (Fig. 3). X-rays showed proximal fragment consolidation and persistent acromioclavicular anatomical reduction (Fig. 4). Ten months after the accident, the patient reports being very satisfied with the overall results.

## 3 Discussion

The first reported case of a floating clavicle, also termed bipolar clavicle injury, or panclavicle injury, dates from late XVII^th^ century [3]. A little over 40 cases have been published ever since, most of them being single-case reports [3,[5], [6], [7], [8], [9], [10], [11], [12], [13], [14]]. This entails a paucity of therapeutic guidelines to aid in decision-making and makes this injury easy to overlook in the ER.

Bipolar clavicle injuries are a rare clinical entity that encompasses a spectrum of clavicle fractures, acromioclavicular or sternoclavicular joint dislocations, with several possible combinations. There is debate regarding the mechanism of injury. A possible explanation is that multidirectional forces are involved, causing a pivoting movement of the clavicle around its midshaft, which is responsible for the usual pattern of proximal anterior and distal posterior displacement. These multidirectional forces are usually the result of a laterally directed blow to the shoulder, combined with a torsional mechanism [5,9]. The reason behind this mechanism is the usual high-energy context in which these injuries are sustained (traffic accidents, sporting injuries, falls from height). Therefore, accompanying injuries occur, with particular clinical relevance of rib fractures, head trauma, and pulmonary contusion, but also other extremity fractures [8]. It is advisable to have a high index of suspicion for floating clavicle in a high-energy trauma patient, given the low incidence but possible concomitant respiratory complications [12].

A bilateral extremity X-ray is recommended to properly account for shortening and elevation of injured clavicle. An AP as well as a serendipity view (30° ascendant X-ray) should be included, to allow for assessment of anteroposterior displacement. The gold standard imaging technique is a computed tomography scan, with 3D reconstruction when available, which will aid in preoperative planning. A further advantage of a CT scan is the possibility to account for other thoracic injuries [9,15].

Despite the lack of management protocols for floating clavicle, most authors agree on surgical management of at least one of the clavicle injuries, with particular emphasis in the young and active patient [7,8,10]. Non-operative treatment has been associated with re-subluxation, residual deformity, persistent pain, and loss of strength of the affected shoulder. However, some authors like Choo et al. [10] argue that it may be preferable to manage the sternoclavicular injury in a conservative manner, given the high surgical risk in such a complex area which is unfamiliar for the orthopedic surgeon [9,10].

Multiple surgical techniques have been published, including management with temporary Kirschner wires, reconstruction plate osteosynthesis [16], hook-plate for the distal clavicle or ACJ dislocation [10], multiple interfragmentary screw synthesis for the proximal clavicle [17], anatomical or non-anatomical joint reconstruction with sutures [8], allo- or autologal ligament graft [11], and transosseous button systems as the one described in this case. Since good results have been reported with all of these techniques, we argue that it is advisable to use the one in which the surgeon is most experienced.

Good results have also been reported in the delayed management of floating clavicle [5,6]. Published cases were due to misdiagnosis or late referral. However, we believe that delayed surgery holds particular interest in the management of the polytrauma patient, who may require clinical stabilization before surgery can be carried out.

## 4 Conclusion

Despite bipolar clavicle fractures being a rare clinical entity, we should suspect them when facing a single clavicle injury in a high-energy trauma context. After an extensive review of the available literature, and the individual surgical indication for isolated injuries in our patient, we opted for surgical treatment. This decision was further encouraged by the fact that he was a young and athletic adult. Our results at 4-month follow-up are very successful, with favorable clinical development, and without radiological loss of reduction. Therefore, we recommend surgical treatment by means of osteosynthesis and soft tissue reconstruction or repair, particularly in the young and high-demand patient. This allows early rehabilitation and return to sport practice. Given the wide diversity of available treatments, we believe it is important to develop guidelines to standardize floating clavicle management.

## Funding

Not applicable.

## Ethical approval

Ethical approval for this report has been exempted by our institution.

## Provenance and peer review

Not commissioned, externally peer-reviewed.

## Consent

Written informed consent was obtained from the patient for publication of this case report and accompanying images. A copy of the written consent is available for review by the Editor-in-Chief of this journal on request. The identity of this patient was protected.

## Author contribution

**Irene Blanca Moreno-Fenoll**: Patient assessment in Emergency Room, Conceptualization, Surgery, Writing - Original Draft, Review & Editing.

**Homero Valencia**: Conceptualization, Surgery, Writing - Review & Editing, Supervision.

**Homid Fahandezh-Saddi**: Conceptualization, Surgery, Writing - Review & Editing.

**Elsa Arruti**: Patient assessment in Emergency Room, Conceptualization, Surgery.

## Registration of research studies

Not applicable.

## Guarantor

Dr. Homero Valencia.

## Declaration of competing interest

We report no conflicts of interest.
